# Exploration of surgical blood pressure management and expected motor recovery in individuals with traumatic spinal cord injury: This article has been corrected since Advance Online Publication and a correction is also printed in this issue

**DOI:** 10.1038/s41393-019-0370-5

**Published:** 2019-10-24

**Authors:** Reza Ehsanian, Jenny Haefeli, Nhung Quach, Jacob Kosarchuk, Dolores Torres, Ellen D. Stuck, Jessica Endo, James D. Crew, Benjamin Dirlikov, Jacqueline C. Bresnahan, Michael S. Beattie, Adam R. Ferguson, Stephen L. McKenna

**Affiliations:** 1 Rehabilitation Research Center, Santa Clara Valley Medical Center, San Jose, CA USA; 2 Department of Neurosurgery, Stanford University, Stanford, CA USA; 3 Division of Physical Medicine and Rehabilitation, Department of Neurosurgery, University of New Mexico School of Medicine, Albuquerque, NM USA; 4 Brain and Spinal Injury Center, Department of Neurosurgery, University of California, San Francisco, San Francisco, CA USA; 5 Eastern Virginia Medical School, Norfolk, VA USA; 6 Department of Physical Medicine and Rehabilitation, Santa Clara Valley Medical Center, San Jose, CA USA; 7 Department of Neurological Surgery, University of California, San Francisco, San Francisco, CA USA

**Keywords:** Spinal cord, Neurophysiology, Spinal cord diseases

## Abstract

**Study design:**

Retrospective analysis.

**Objective:**

To assess the impact of mean arterial blood pressure (MAP) during surgical intervention for spinal cord injury (SCI) on motor recovery.

**Setting:**

Level-one Trauma Hospital and Acute Rehabilitation Hospital in San Jose, CA, USA.

**Methods:**

Twenty-five individuals with traumatic SCI who received surgical and acute rehabilitation care at a level-one trauma center were included in this study. The Surgical Information System captured intraoperative MAPs on a minute-by-minute basis and exposure was quantified at sequential thresholds from 50 to 104 mmHg. Change in International Standards for Neurological Classification of Spinal Cord Injury (ISNCSCI) motor score was calculated based on physiatry evaluations at the earliest postoperative time and at discharge from acute rehabilitation. Linear regression models were used to estimate the rate of recovery across the entire MAP range.

**Results:**

An exploratory analysis revealed that increased time within an intraoperative MAP range (70–94 mmHg) was associated with ISNCSCI motor score improvement. A significant regression equation was found for the MAP range 70–94 mmHg (F[1, 23] = 4.65, *r*^2^ = 0.168, *p* = 0.042). ISNCSCI motor scores increased 0.036 for each minute of exposure to the MAP range 70–94 mmHg during the operative procedure; this represents a significant correlation between intraoperative time with MAP 70–94 and subsequent motor recovery. Blood pressure exposures above or below this range did not display a positive association with motor recovery.

**Conclusions:**

Hypertension as well as hypotension during surgery may impact the trajectory of recovery in individuals with SCI, and there may be a direct relationship between intraoperative MAP and motor recovery.

## Introduction

Estimates of the annual global incidence of spinal cord injury (SCI) range from 40 to 80 cases per million [1–3]. There are approximately 17,700 new cases each year in the United States [4]. SCI results in significant functional impairment, loss of independence, morbidity, mortality, and high lifetime costs, highlighting the importance of limiting the cascade of damage to the microenvironment around the injury [5]. Progress has been made in understanding the primary and secondary mechanisms of injury that damage the spinal cord [6]. It is hypothesized that interventions that limit the secondary injury process, such as limiting hypotension and spinal cord ischemia, may improve individual outcomes [7]. Our group has proposed physiological mechanisms involving a complex and highly interrelated series of molecular processes such as ionic dysregulation, free radical production, cytoskeletal degradation, and neuroinflammation [8]. In addition, a topological analysis of preclinical data by our group demonstrated that the occurrence of intraoperative hypertension in a preclinical model may also result in impaired functional and neurological recovery [9]. Therefore, this study was undertaken to assess the potential clinical impact of intraoperative blood pressure variability during surgical stabilization following traumatic SCI.

In the 1970s, blood pressure became a target of systematic augmentation for individuals with traumatic SCI. There have been many efforts to generate quality data that specify an optimal mean arterial pressure (F) range. However, these studies were limited by lack of comparison groups or suffer from other confounding elements [10–12]. Current guidelines for blood pressure augmentation in traumatic SCI are largely based on the 1997 study by Vale and associates [13]. The Congress of Neurological Surgeons (CNS) and the American Association of Neurological Surgeons (AANS) (CNS/AANS) issued updated guidelines in 2013 that recommend maintaining MAP between 85 and 90 mmHg in the first 5−7 days after a traumatic SCI in order to improve cord perfusion [14]. These guidelines were affirmed in a recent meta-analysis by Saadeh et al. in 2017 [15]. Although these postinjury MAP goals are widely accepted, there is a paucity of evidence to support this practice [14–16]. Furthermore, alternatives to the 85–90 mmHg MAP range have been proposed [8, 17–21]. Taken together, the current available evidence indicates a continued need for studies to inform MAP augmentation targets that maximize neurologic recovery following traumatic SCI. Previously our group demonstrated that MAP values correlated with improved recovery in the first 2–3 days postinjury, but decreased in significance over the first 5–7 days after injury, leading us to investigate the impact of intraoperative MAP management [19]. The aim of this retrospective study was to assess how intraoperative MAP during spinal surgery may relate to recovery following traumatic SCI as measured by the International Standards for Neurological Classification of SCI (ISNCSCI) motor score.

## Methods

### Study population

Santa Clara Valley Medical Center (SCVMC) is home to both a Level 1 Trauma Center and a Commission on Accreditation of Rehabilitation Facilities (CARF)-accredited Rehabilitation Center serving Northern California. The study population was determined based on individuals admitted at SCVMC Trauma Service with atraumatic SCI, received spinal stabilization surgery and were admitted to the Rehabilitation Center at SCVMC, with postsurgical and discharge from acute rehabilitation ISNCSCI examinations. Individuals were excluded from the study if ISNCSCI examination data were unclear or absent upon chart review or if there was a preexisting degenerative neuromuscular disease. When an individual was deemed to fit the inclusion and exclusion criteria for the study, electronic and paper medical records and intra-operative MAP values were obtained; intraoperative MAP values were digitally obtained from Surgical Information Systems (Alpharetta, GA, USA). Data abstracted from medical records included individual age, sex, change in ISNCSCI motor score, manually recorded MAP measurements throughout surgery, hospital care days, acute rehabilitation days, postsurgery and discharge from acute rehabilitation ISNCSCI examinations, mechanism of injury, and type of vasopressor agents used during surgery to maintain MAP goals as per our center’s standard of care. Ethics approval was obtained from the Institutional Review Board to conduct a retrospective chart review from May 2013 to September 2015 of admission records from SCVMC.

### Surgical mean arterial pressure

Intraoperative MAP values were obtained by an arterial line blood pressure monitor. The duration of blood pressure monitoring was based on medical necessity as judged by the treating anesthesiologist. MAP data were collected at 1-min intervals for each individual by the Surgical Information Systems (SIS). Electronic data were exported from the SIS, Structured Query Language (SQL) database, de-identified and imported into MATLAB version R2016b (MathWorks Inc., Natick, MA, USA) for analysis.

### Automated filter and mean arterial pressure binning

The electronic intraoperative MAP data were first reviewed and validated to exclude nonphysiologic readings before the start and after the end of surgery by the investigators using the manually recorded anesthesia record (clinical curated data). These validated data were compared to the manual anesthesiologist MAP recordings and systematically reviewed by three clinicians (RE, SLM, NQ). Based on insights of the reviewing clinicians and investigators into invalid data (e.g., motion artifacts and injections) exported from the SIS SQL database, a heuristic was created for an automated filter using MATLAB. The MATLAB filter removed MAP values under 10 and above 200 as well as point-to-point changes greater than 40, as these instances were found to represent data artifacts. Inter-rater reliability was calculated between the clinical curated data and the MATLAB filtered data. The automated filter produced an array that was 99.1% accurate with a sensitivity of 99.5% and specificity of 93.2% on average for all individuals. Upon review of the differences between the clinical curated data and automated filtering, it was found that automated filtering more accurately identified invalid data. Therefore, MATLAB filtered data were used for the analysis presented below.

After filtering each individual’s MAP data, 5 mmHg unit bins were created to assess the total amount of time the individual experienced MAP values within this range or below a threshold. For instance, for binning MAP values within a range, a “countIF” statement was used to count the number of instances a filtered MAP value fell between a 5-unit ranges (e.g., 50–54 mmHg). In this way, eleven 5 mmHg unit filtered MAP bins (50–104 mmHg) were created. Filtered MAP values were distributed into 11 groups (ranges): 50–54, 55–59, 60–64, 65–69, 70–74, 75–79, 80–84, 85–89, 90–94, 95–99, and 100–104. Additionally, as part of a secondary analysis, the data were divided into time spent in hypotensive (MAP 50–69), normal/optimal (MAP 70–94), and hypertensive (MAP 95–104) states. These thresholds were determined based on the regression analysis (see Discussion for additional rationale).

### Outcome measure: International Standards for Neurological Classification of SCI (ISNCSCI) motor scores

The primary outcome measure of the study is the ISNCSCI motor score [22]. Individual outcome data were abstracted from the electronic medical record and included exam data at the earliest postoperative and discharge from acute rehabilitation examinations.

### Statistical analysis

Due to the complex nature of SCI pathophysiology, we developed an analytical workflow according to the same statistical principals as our previous methods to account for the heterogeneity of this disorder [9, 23–26]. For the current study, the workflow begins with a sequential evaluation of motor recovery in comparison to time within a predefined series of blood pressure parameters, which is similar to previous studies [17, 19]. This allows for visualizing the relationship between changes in ISNCSCI motor score versus time spent within defined MAP range. The data from this analysis can then be used to analyze the relationship between changes in ISNCSCI motor score versus time spent within optimal (positive slope) versus nonoptimal (negative slope) MAP ranges. Analysis was performed using SPSS software (24 for Windows; SPSS Inc.) and Prism (7.02 for Windows, GraphPad Software). To investigate group differences in demographic information as well as injury and surgical characteristics between individuals that increased their ISNCSCI motor scores (improvement group) and those that did not (no improvement group), Fisher’s exact tests and Mann−Whitney *U* tests were used. The no-improvement group included individuals who either showed no change or decline in the ISNCSCI motor scores. The improved group included individuals who improved at least one point in the ISNCSCI motor score. Fisher’s exact tests were used for categorical data (sex, AIS grade, mechanism of injury, level of injury, and number of vasopressors), while Mann−Whitney *U* tests were done for continuous data (age, time to surgery, surgical time, MAP mean, inpatient length of stay, and acute rehabilitation length of stay and time between ISNCSCI).

To investigate the association of MAP and motor score changes, a series of linear regressions were employed. Linear regressions using ISNCSCI motor score change (rehabilitation discharge − post-surgery) (dependent variable) versus minutes within MAP bin (independent variable) were fit across 11 MAP bins. Changes in beta values (regression slopes) were used to visualize differences in the associations within each linear regression. Specifically, the investigators were interested in observing instances where the beta values changed from negative to positive and then back to negative, as these changes may align with potential deleterious clinical consequences of hypotensive and hypertensive states during surgery.

The optimal MAP range identified by this exploratory analysis (MAP 70–94 mmHg) was then used in a secondary analysis to investigate the association between ISNCSCI motor score changes and exposure time within and outside of this optimal range. Mann−Whitney *U* tests were employed to compare two groups (ISNCSCI motor score improvement and no improvement) on the duration (minutes) spent out of the optimal range as defined by the CNS/AANS guidelines for MAP management in SCI (MAP 85–90 mmHg) as well as the optimal MAP range identified in the regression analysis (MAP 70–94 mmHg).

## Results

### Individual characteristics

Twenty-five individuals had available digitally collected MAP data and ISNCSCI motor scores. Individuals were divided into two groups, no improvement and improvement, based on the change in ISNCSCI motor score (Table 1). In the study cohort there were 16 individuals with improvement and 9 individuals with no improvement. In the no-improvement group, three individuals had worsening results. No significant differences were observed between group’s characteristics (*p* > 0.16; Table 1).

**Table 1:** Individual characteristics. This table summarizes the individual characteristics for individuals who improved and did not improve ISNCSCI motor scores

Characteristics	No improvement (*n* = 9)	Improvement (*n* = 16)	MW-U	*p* values
Age (yrs)	42.7 (17.9)	42.3 (18.1)	65.5	0.71
Time to surgery (h)	39.6 (29.8)	49.6 (25.6)	64.0	0.65
Surgery time (min)	431 (126)	405 (188)	56.0	0.37
MAP mean (mmHg)	76.1 (7.27)	79.5 (5.94)	47.0	0.16
Inpatient LOS (days)	60.8 (31.6)	48.9 (29.5)	56.5	0.38
Acute rehab LOS (days)	35.4 (25.5)	29.0 (13.6)	66.5	0.76
Time between ISNCSCI (days)	41.8 (29.8)	52.4 (25.6)	59.5	0.48
Sex			^a^	0.39
Male	5 (55.6)	12 (75)		
Female	4 (44.4)	4 (25)		
AIS Grade			^a^	0.46
AIS A	3 (33.3%)	8 (50.0%)		
AIS B	3 (33.3%)	1 (6.30%)		
AIS C	1 (11.1%)	2 (12.5%)		
AIS D	2 (22.2%)	5 (31.3%)		
Mechanism of injury			^a^	0.50
Sports	3 (33.3%)	5 (31.3%)		
Transport	1 (11.1%)	5 (31.3%)		
Fall	4 (44.4%)	6 (37.5%)		
Other traumatic	1 (11.1%)	0 (0.00%)		
Level of injury			^a^	0.63
Cervical	6 (66.7%)	13 (81.3%)		
Thoracic	3 (33.3%)	3 (18.8%)		
Vasopressors^b^			^a^	0.60
One vasopressors	2 (22.2%)	2 (12.5%)		
Two vasopressors	3 (33.3%)	8 (50.0%)		
Three vasopressors	3 (33.3%)	5 (31.3%)		
Four vasopressors	1 (11.1%)	0 (0.00%)		

Continuous variables are summarized with means and standard deviations (in parentheses), while categorical variables are summarized with counts and ranges (in parentheses)

*MW-U* Mann−Whitney *U* test, *yrs* years, *h* hours, min minutes, *MAP* mean arterial pressure, *LOS* length of stay, *AIS* American Spinal Injury Association Impairment Scale

^a^Fisher’s exact test

^b^The record of vasopressor administration was missing for one individual in the improvement group

The majority of individuals required intraoperative vasopressors to maintain MAP (Table 1 and Supplementary Table 1). All individuals in the no-improvement group required vasopressor agents; seven of these individuals required combinations of two or more vasopressor agents to maintain MAP goals. In the improvement group 15 individuals required vasopressor agents to achieve targeted MAP, and 13 of these individuals required different combinations of two or more vasopressor agents (Table 1 and Supplementary Table 1). The record of vasopressor administration was missing for one individual in the improvement group.

### Relationship between changes in ISNCSCI motor score versus time spent within defined MAP range

**Fig. 1:**
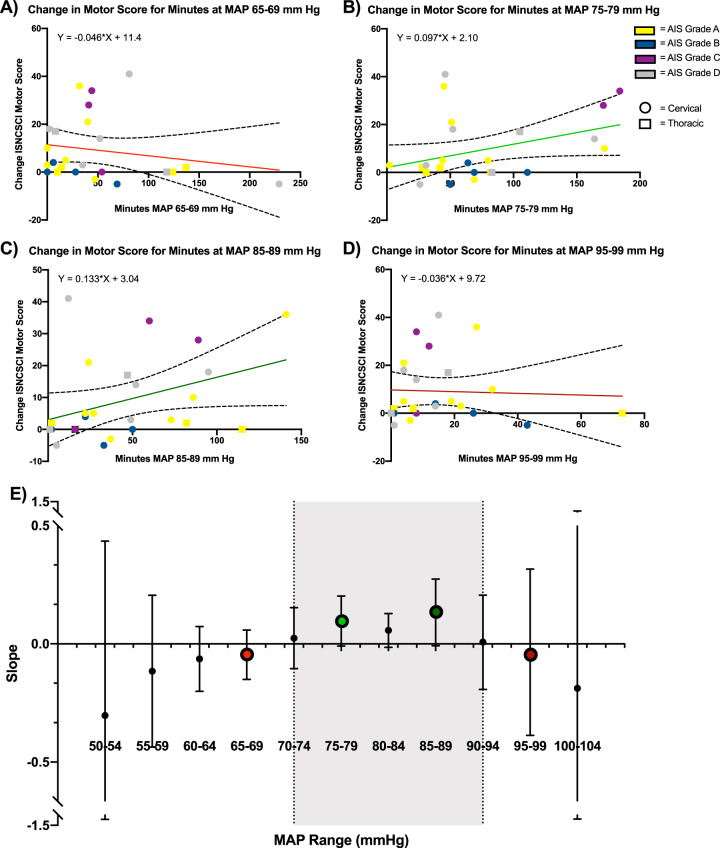
Serial regression analysis between MAP exposure and ISNCSCI motor score changes. Representative figures (**a**–**d**) illustrate individual regressions between 5-unit MAP bins and changes in ISNCSCI motor scores. A trend line, confidence intervals, and trend line equation are included in the representative figures. Each point in the representative figures corresponds to an individual. Linear regressions with positive slope (green) signify greater improvement in motor score based on increased exposure within a defined MAP range. Negative slopes (red) signify greater improvement in motor score based on decreased exposure within a defined MAP range. A negative slope does not represent deterioration of individual motor scores. **e** summarizes the 11 individual 5-unit MAP bin regressions. The *x* axis represents the MAP bins and the *y* axis represents the slopes (betas) for the individual regressions. Each point includes its corresponding 95% confidence interval, illustrated as an error bar. The gray region represents the optimal MAP range identified in this analysis

Following SCI, motor function as measured by the ISNCSCI is expected to improve [27]. Improvements are noted to take place over the course of approximately 1 year with most of the motor improvement occurring within months of injury. Eleven individual regressions were completed to analyze the association between a 5-unit MAP range and ISNCSCI motor score changes. Graphical representations of the individual regressions are presented in Fig. 1a–d. In order to facilitate interpretation of the relationship between MAP and motor score improvement across all 11 linear regressions (Supplementary Table 2), the slopes of the individual models were plotted on a composite graph (Fig. 1e). Positive values represent increased motor score improvement based on increased exposure within an MAP range; conversely, negative values represent increased motor score improvement based on decreased exposure within an MAP range. The linear regression fit to each data set demonstrates that individuals segregate, such that time exposure within MAP 70 mmHg to 94 mmHg display increased motor score improvement based on increased exposure within the 70–94 mmHg MAP range.

### Relationship between changes in ISNCSCI motor score versus time spent within optimal versus nonoptimal MAP range

**Fig. 2:**
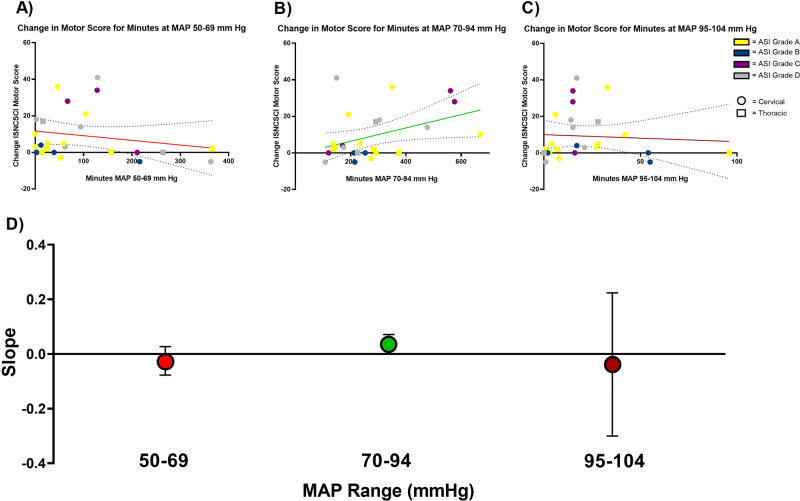
Change of ISNCSCI motor score vs. minutes within each MAP range during surgery. **a**–**c** Linear regression of ISNCSCI motor scores change (discharge—post-surgery) vs. minutes within each MAP range during surgery; each point represents an individual. Positive slopes (green) signify greater improvement in motor score based on increased exposure within a defined MAP range. Negative slopes (red) signify greater improvement in motor score based on decreased exposure within a defined MAP range. A negative slope does not represent deterioration of individual motor scores. **d** Graph of slopes for each linear regression analysis; each point represents the slope of regression line. Colored points correspond to slopes of (**a**−**c**) and error bars correspond to the 95% confidence interval of the slope (beta)

To investigate the relationship between motor improvement and time spent within the MAP range 70–94 mmHg, linear regressions were conducted for MAP ranges of 50–69, 70–94, and 95–104 mmHg (Fig. 2 and Supplementary Table 3). The beta coefficient for the linear regression modeling change in motor score versus time exposure to 70–94 mmHg MAP range was 0.036 (CI: 0.001–0.071, *p* = 0.042) (Fig. 2b), representing a positive association. Beta coefficients for both the hypotension (50–69 mmHg; beta: −0.025, CI: −0.077 to 0.027, *p* = 0.322) and the hypertension (95–104 mmHg; beta: −0.039, CI: −0.301 to 0.224, *p* = 0.764) MAP ranges showed a negative association but did not reach statistical significance. The intercept for the normotensive model was −0.880, representing the starting ISNCSCI motor score change before time exposure to the 70–94 mmHg MAP range. The intercepts for the hypotension and hypertension models were 11.7 and 10.0 ISNCSCI motor points, respectively. Figure 2d displays the transition from increased motor score improvement based on increased time exposure within the 70–94 mmHg MAP range; conversely, negative values were observed for time exposure within the hypotensive and hypertensive MAP range.

### Relationship between time within optimal MAP range and motor score improvement

**Fig. 3:**
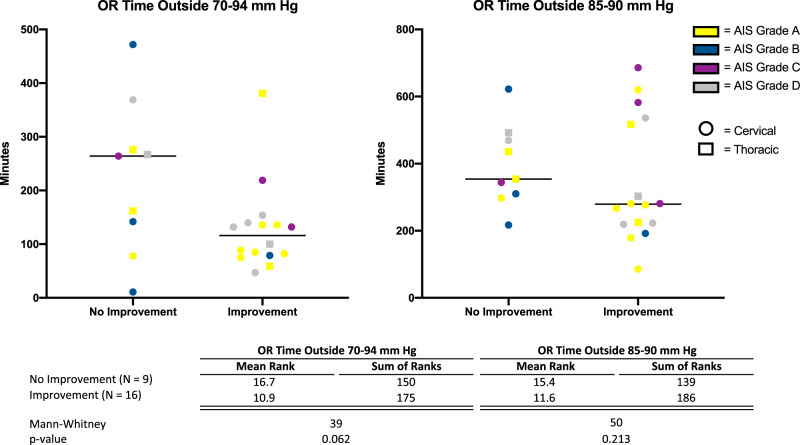
Surgical time outside 70–94 MAP range and 85–90 MAP range for individuals with no improvement versus individuals with improvement. Each figure represents the distribution of time (minutes) spent outside the optimal (70–94 mmHg) and CNS/AANS (85–90 mmHg) recommended MAP ranges for the improvement and no improvement groups. The figure on the left represents the optimal range identified in the regression analysis, while the figure on the right represents the CNS/AANS-recommended range. Each point represents an individual and the lines represent the median exposure time for the group

To investigate the impact of blood pressure on motor score improvement, nonparametric tests were utilized. Results from the Mann−Whitney *U* tests (Fig. 3) showed no differences in the time (minutes) spent outside of the CNS/AANS guidelines for MAP management in SCI (MAP 85–90) between the ISNCSCI motor score improver (median exposure time = 279) and nonimprover groups (median exposure time = 354; *U* = 50, *p* = 0.213). However, utilizing the optimal (70–94 mmHg) MAP range identified within the regression, individuals who improved tended to spend less time outside this range (median exposure time = 77.5) compared to the nonimprovers with the test approaching statistical significance (median exposure time = 161; *U* = 39, *p* = 0.062).

## Discussion

This paper presents a systematic analysis of the impact of operative blood pressure management on motor recovery following SCI during the acute inpatient rehabilitation phase of care. The majority of individuals with SCI will experience some degree of spontaneous motor recovery [27]. When motor recovery is observed, it is most significant during the first 3–6 months following SCI; however, the meaning of increases of motor score may reflect differences between thoracic and cervical spinal cord injury. The former represents long tract function only, and the latter includes segmental function [27, 28]. The effect of blood pressure management following SCI has been studied for decades based on the belief that early augmentation of MAP may improve initial outcomes at the time of discharge from inpatient hospitalization [13]. This study extends the window of analysis beyond discharge from hospitalization to discharge from inpatient rehabilitation. Previous work from our group demonstrated an ICU MAP range that corresponded with optimal motor recovery [8, 17, 19]. The current analysis presented here focuses specifically on intraoperative MAP exposure and exploring the deleterious effects of both hypotension, as well as hypertension, on motor recovery.

### Optimal MAP range to promote neurological motor recovery

The results of the current study support recommendations for maintaining a narrow MAP range during the acute phase of SCI care, specifically during initial spinal stabilization surgery, with attention to both hypotension as well as hypertension. In this study, we used a threshold-based analytic strategy to evaluate the relationship between MAP and motor recovery. This study observed that greater time spent within an MAP range of 70–94 mmHg was significantly associated with greater ISNCSCI motor score changes. As demonstrated previously by our group the difficulty of interpreting these data may be overcome by a graphical representation over a range of potential blood pressure thresholds [19]. This study provides preliminary evidence supporting an association between MAP values during operative management of SCI and motor recovery. It does not, however, provide evidence of a causal relationship.

Of potential clinical relevance, this association between MAP and motor recovery appeared to be the case even for those initially with complete neurological injury. Of the eleven AIS A individuals in this study, eight showed motor improvement (Fig. 1). Further, seven of these eight AIS A individuals who improved were noted to have spent less time in the OR outside of the MAP 70–94 mmHg range than the majority of those who did not show motor recovery (Fig. 3). Conversely, two of the seven with the least neurologically complete SCI (AIS D) did not demonstrate motor recovery during the study time period (Fig. 1). Each of the two AIS D individuals who did not show motor improvement were found to have spent more time in the OR outside the MAP 70–94 mmHg range than the majority of those who did not improve (Fig. 3). Taken together, these observations within the AIS A and AIS D groups further suggest tight BP control in the OR within a range of 70–94 mmHg may enhance early motor recovery. If true, this would suggest tight BP control is critical for potential motor recovery, regardless of the neurological completeness of SCI. While interesting and potentially clinically important, these observations would need to be validated by a larger prospective study.

### Hypotension detrimental for neurological motor recovery

In 1984, Tator and his associates found that neurological and mortality outcomes were improved with early intensive care unit (ICU) management and avoidance of hypotension [29]. The current CNS/AANS guidelines for acute SCI blood pressure management differ from broadly defined definitions of hypotension for the majority of ICU settings [30]. A review by Hylands et al. of vasopressor blood pressure targets in critically ill adults supported the target of 60 mmHg and demonstrated MAPs greater than 70 mmHg did not correlate with increased benefit [31]. However, in the critical care setting for patients with SCI, Cohn et al. and Hawryluk et al. both found that MAPs below a threshold of 70 mmHg correlated with worse outcomes [17, 19]. Specifically, Cohn showed that SCI patients admitted to the ICU who spent increasing time with MAPs below thresholds 70 mmHg experienced lower total motor score change from admission to discharge from rehabilitation. In the present study, the lowest MAP at which individuals’ improvement were distinguished from those with optimal motor recovery was 70 mmHg, suggesting that this may be the lowest blood pressure threshold associated with motor recovery. Moreover, data from the current study suggest that MAP ranges above 94 mmHg may be detrimental to the spontaneous recovery of motor function.

### Hypertension detrimental for neurological motor recovery

The results of the current study display a trend suggesting that MAP greater than 94 mmHg may also be associated with failure to achieve the motor recovery observed for the 70–94 mmHg MAP range. Animal models demonstrate that normotension should be maintained and that induced hypertension avoided given the evidence suggesting increased risk of spinal cord hemorrhage [32]. Subsequent animal models of the risk of hypertension demonstrated that norepinephrine did not improve spinal cord perfusion but was associated with increased size of parenchymal hemorrhage [33]. Kepler et al. and Inoue et al. found either decreased motor function with MAP > 85 mmHg or no correlation between MAP > 85 mmHg and motor recovery [34, 35]. Given the evidence that maintenance of hypertension may have deleterious effects, Kwon et al. evaluated clinical equipoise for motor outcomes among patients randomized to spinal cord perfusion pressure (SCPP) ≥ 75 mmHg or avoidance of hypotension with MAP ≥ 65 mmHg [36]. In light of this clinical equipoise, a phase III clinical trial is underway (NCT02232165) to evaluate the noninferiority of MAP ≥ 65 mmHg vs. ≥85 mmHg in patients with SCI. The current study lends evidence to the objective of maintaining blood pressure below 94 mmHg.

### Study limitations

The main limitations of this study are the small sample size (*n* = 25) and retrospective nature of the study. Because of the small sample size and heterogeneity of the data, the individual regression analyses were not sufficiently powered to achieve statistical significance. Another limitation of the analysis is the setting of blood pressure monitoring that was focused on data collected while the individual was undergoing spinal stabilization. Because of the limitation in availability of pre-decompression data, we have not addressed the potential effect of exposure to significant variation in MAP in the ambulance and emergency department [37]. Finally, recent advances in intraspinal pressure monitoring have suggested that spinal cord perfusion pressure (SCPP) may be more accurate, improve predictions of recovery, and be more sensitive to the effect of interventions [38].

## Conclusion

This study suggests a deviation from a range of mean arterial blood pressure between 70 and 94 mmHg during the operative management of SCI may affect motor recovery. It is important to note that most individuals’ motor scores did not change and only a few worsened. Hypotension as well as hypertension in the acute surgical setting for individuals with SCI may impact the trajectory of motor recovery.

### Clinical relevance

This study is clinically relevant as it provides evidence to support the utility of MAP goals to limit both hypotension and hypertension in early management of traumatic SCI.

### Data archiving

All relevant raw data will be freely available to any researcher wishing to use them for noncommercial purposes, without breaching individual confidentiality.

## Supplementary information

Supplemental Tables
