# Correction: Exploration of surgical blood pressure management and expected motor recovery in individuals with traumatic spinal cord injury

**DOI:** 10.1038/s41393-019-0400-3

**Published:** 2019-12-10

**Authors:** Reza Ehsanian, Jenny Haefeli, Nhung Quach, Jacob Kosarchuk, Dolores Torres, Ellen D. Stuck, Jessica Endo, James D. Crew, Benjamin Dirlikov, Jacqueline C. Bresnahan, Michael S. Beattie, Adam R. Ferguson, Stephen L. McKenna

**Affiliations:** 1 Rehabilitation Research Center, Santa Clara Valley Medical Center, San Jose, CA USA; 2 Department of Neurosurgery, Stanford University, Stanford, CA USA; 3 Division of Physical Medicine and Rehabilitation, Department of Neurosurgery, University of New Mexico School of Medicine, Albuquerque, NM USA; 4 Brain and Spinal Injury Center, Department of Neurosurgery, University of California, San Francisco, San Francisco, CA USA; 5 Eastern Virginia Medical School, Norfolk, VA USA; 6 Department of Physical Medicine and Rehabilitation, Santa Clara Valley Medical Center, San Jose, CA USA; 7 Department of Neurological Surgery, University of California, San Francisco, San Francisco, CA USA

**Keywords:** Spinal cord, Neurophysiology, Spinal cord diseases

**Correction to: Spinal Cord**

10.1038/s41393-019-0370-5

published online 24 October 2019

In the original version of this article, one participant’s sex was assigned incorrectly. In Table 1, the number of males in the ‘Improvement’ group was misstated as ‘13 (81.3)’, instead of ‘12 (75)’, and the number of females was misstated as ‘3 (18.8)’, instead of ‘4 (25)’. As a result, the associated *p*-value has changed from ‘0.21’ to ‘0.39’. This has been corrected in both the PDF and HTML versions of the article.

