# Correction: Exploration of surgical blood pressure management and expected motor recovery in individuals with traumatic spinal cord injury

**DOI:** 10.1038/s41393-020-0504-9

**Published:** 2020-06-23

**Authors:** Reza Ehsanian, Jenny Haefeli, Nhung Quach, Jacob Kosarchuk, Dolores Torres, Ellen D. Stuck, Jessica Endo, James D. Crew, Benjamin Dirlikov, Jacqueline C. Bresnahan, Michael S. Beattie, Adam R. Ferguson, Stephen L. McKenna

**Affiliations:** 1 Rehabilitation Research Center, Santa Clara Valley Medical Center, San Jose, CA USA; 2 Department of Neurosurgery, Stanford University, Stanford, CA USA; 3 Division of Physical Medicine and Rehabilitation, Department of Neurosurgery, University of New Mexico School of Medicine, Albuquerque, NM USA; 4 Brain and Spinal Injury Center, Department of Neurosurgery, University of California, San Francisco, San Francisco, CA USA; 5 Eastern Virginia Medical School, Norfolk, VA USA; 6 Department of Physical Medicine and Rehabilitation, Santa Clara Valley Medical Center, San Jose, CA USA; 7 Department of Neurological Surgery, University of California, San Francisco, San Francisco, CA USA

**Keywords:** Spinal cord, Neurophysiology, Spinal cord diseases

Correction to: *Spinal Cord* 58:377–386

10.1038/s41393-019-0370-5 published online 24 October 2019

Following publication of this article, an error was identified in the data structure for the analysis “Relationship between changes in ISNCSCI motor score versus time spent within optimal versus nonoptimal MAP range”. The published article has now been corrected, and the changes are detailed below:

(1) In the “Abstract”:

“…(*F*[1, 23] = **5.07**, *r*^2^ = **0.181**, *p* = **0.034**). ISNCSCI motor scores increased **0.039** for each minute of exposure to the MAP range 70–94 mmHg during the operative procedure.”

was changed to:

“…(*F*[1, 23] = **4.65**, *r*^2^ = **0.168**, *p* = **0.042**). ISNCSCI motor scores increased **0.036** for each minute of exposure to the MAP range 70–94 mmHg during the operative procedure.”

(2) In the “Methods”, under the heading “Automated filter and mean arterial pressure binning”:

“Additionally, as part of a secondary analysis, the data were divided into time spent in hypotensive **(MAP < 70)**, normal/optimal **(MAP between 70 and 94)**, and hypertensive **(MAP > 94)** states.”

was changed to:

“Additionally, as part of a secondary analysis, the data were divided into time spent in hypotensive **(MAP 50–69)**, normal/optimal **(MAP 70–94)**, and hypertensive **(MAP 95–104)** states.”

(3) In the “Results”, under the heading “Relationship between changes in ISNCSCI motor score versus time spent within optimal versus nonoptimal MAP range”:

“The beta coefficient for the linear regression modeling change in motor score versus time exposure to 70–94 mmHg MAP range was **0.039 (CI: 0.003–0.076,** ***p* = 0.034)** (Fig. 2b), representing a positive association. Beta coefficients for both the hypotension (50–69 mmHg; beta: −0.025, **CI: −0.074 to 0.025,** ***p* = 0.311**) and the hypertension (95–104 mmHg; **beta: −0.013, CI: −0.112 to 0.085,** ***p* = 0.782**) MAP ranges showed a negative association but did not reach statistical significance. The intercept for the normotensive model was **−0.664**, representing the starting ISNCSCI motor score change before time exposure to the 70–94 mmHg MAP range. The intercepts for the hypotension and hypertension models were **11.8 and 9.99** ISNCSCI motor points, respectively.”

was changed to:

“The beta coefficient for the linear regression modeling change in motor score versus time exposure to 70–94 mmHg MAP range was **0.036 (CI: 0.001–0.071,** ***p* = 0.042)** (Fig. 2b), representing a positive association. Beta coefficients for both the hypotension (50–69 mmHg; beta: −0.025, **CI: −0.077 to 0.027,** ***p* = 0.322**) and the hypertension (95–104 mmHg; **beta: −0.039, CI: −0.301 to 0.224,** ***p* = 0.764**) MAP ranges showed a negative association but did not reach statistical significance. The intercept for the normotensive model was **−0.880**, representing the starting ISNCSCI motor score change before time exposure to the 70–94 mmHg MAP range. The intercepts for the hypotension and hypertension models were **11.7 and 10.0** ISNCSCI motor points, respectively.”

(4) Fig. 2 has also been corrected. The original, incorrect version of Fig. 2 is displayed below:



(5) Finally, the supplementary table file has been updated to include the corrected version of table S3. The original, incorrect version is displayed below:

**Table S3:** Linear Regressions of ISNCSCI Motor Score vs. Minutes within each MAP range during surgery.

MAP Range (mm Hg)	Equation	95% CI			Goodness of Fit		Is slope significantly non-zero?			
		Slope	Y-intercept	X-intercept	R Square	Sy.X	F	DFn, Dfd	P Value	Deviation from Zero?
50-69	Y = -0.025*X + 11.8	-0.074 to 0.025	4.23 to 19.3	193 to +infinity	0.045	13.4	1.07	1, 23	0.311	Not Significant
70-94	Y = 0.039*X - 0.664	0.003 to 0.076	-11.1 to 9.74	-265 to 168	0.181	12.4	5.08	1, 23	0.034	Significant
95-104	Y = -0.013*X + 9.99	-0.112 to 0.086	1.86 to 18.1	122 to +infinity	0.003	13.7	0.078	1, 23	0.782	Not Significant

