# Supplementary material for: Exploration of surgical blood pressure management and expected motor recovery in individuals with traumatic spinal cord injury: This article has been corrected since Advance Online Publication and a correction is also printed in this issue
Source: Spinal Cord. 2019 Oct 24;58(3):377–86. doi: 10.1038/s41393-019-0370-5 (PMC7062632; doi:10.1038/s41393-019-0370-5)
Supplement: Supplementary file 1 — Supplemental Tables [file 41393_2019_370_MOESM1_ESM.pdf]

Supplementary Table 1. Administered Operating Room Vasopressors

Participant	Vasopressors
1	Phenylephrine, Ephedrine
2	Dopamine, Norepinephrine, Phenylephrine
3	Phenylephrine
4	Vasopressin, Norepinephrine
5	Phenylephrine, Ephedrine
6	Dopamine, Phenylephrine, Ephedrine
7	Phenylephrine
8	Dopamine, Phenylephrine, Ephedrine
9	Dopamine, Phenylephrine, Ephedrine
10	Dopamine, Phenylephrine, Ephedrine
11	Phenylephrine, Ephedrine
12	Phenylephrine, Ephedrine
13	Phenylephrine, Ephedrine
14	Phenylephrine, Ephedrine, Epinephrine,
15	No OR med records
16	Dopamine, Phenylephrine, Ephedrine
17	Dopamine, Phenylephrine, Ephedrine
18	Phenylephrine, Vasopressin
19	Phenylephrine, Ephedrine, Vasopressin
20	Phenylephrine
21	Phenylephrine, Ephedrine
22	Phenylephrine, Ephedrine
23	Phenylephrine, Ephedrine
24	Phenylephrine
25	Phenylephrine, Ephedrine

Supplementary Table 2. Linear Regressions of ISNCSCI Motor Score vs. Minutes within Each MAP Range during Surgery.

MAP Range (mm Hg)	Equation	95% CI			Goodness of Fit		Is slope significantly non-zero?			
		Slope	Y-intercept	X-intercept	R Square	Sy.X	F	DFn, Dfd	P Value	Deviation from Zero?
50-54	Y = -0.303*X + 10.7	-1.04 to 0.434	4.03 to 17.5	11.4 to +infinity	0.030	13.5	0.722	1, 23	0.404	Not Significant
55-59	Y = -0.116*X + 10.9	-0.437 to 0.205	3.63 to 18.1	29.5 to +infinity	0.024	13.5	0.558	1, 23	0.463	Not Significant
60-64	Y = -0.061*X + 11.2	-0.201 to 0.073	4.20 to 18.1	63.2 to +infinity	0.039	13.4	0.931	1, 23	0.345	Not Significant
65-69	Y = -0.046*X + 11.4	-0.151 to 0.058	3.92 to 18.9	91.9 to +infinity	0.035	13.4	0.837	1, 23	0.370	Not Significant
70-74	Y = 0.024*X + 7.84	-0.105 to 0.153	-1.38 to 17.1	-infinity to 11.3	0.006	13.6	0.148	1, 23	0.704	Not Significant
75-79	Y = 0.097*X + 2.10	-0.009 to 0.202	-7.26 to 11.5	-infinity to 42.3	0.136	12.7	3.61	1, 23	0.070	Not Significant
80-84	Y = 0.057*X + 4.98	-0.015 to 0.128	-2.56 to 12.5	-infinity to 25.2	0.105	12.9	2.70	1, 23	0.114	Not Significant
85-89	Y = 0.133*X + 3.04	-0.008 to 0.274	-5.32 to 11.4	-infinity to 23.4	0.143	12.7	3.83	1, 23	0.063	Not Significant
90-94	Y = 0.007*X + 9.02	-0.193 to 0.206	1.27 to 16.8	-infinity to -8.27	<.001	13.7	0.005	1, 23	0.945	Not Significant
95-99	Y = -0.036*X + 9.72	-0.387 to 0.316	2.07 to 17.4	33.0 to +infinity	0.002	13.7	0.044	1, 23	0.836	Not Significant
100-104	Y = -0.188*X + 10.4	-1.02 to 0.642	2.70 to 18.1	13.1 to +infinity	0.010	13.6	0.220	1, 23	0.644	Not Significant

Supplementary Table 3. Linear Regressions of ISNCSCI Motor Score vs. Minutes within each MAP range during surgery.

MAP Range (mm Hg)	Equation	95% CI			Goodness of Fit		Is slope significantly non-zero?			
		Slope	Y-intercept	X-intercept	R Square	Sy.X	F	DFn, Dfd	P Value	Dviation from Zero?
50-69	Y = -0.02543*X + 11.69	-0.07746 to 0.02659	4.164 to 19.23	182.4 to +infinity	0.04257	13.38	1.023	1, 23	0.3224	Not Significant
70-94	Y = 0.03636*X - 0.8802	0.001477 to 0.07125	-11.84 to 10.08	-5994 to 189.3	0.1682	12.47	4.649	1, 23	0.0418	Significant
95-104	Y = -0.03851*X + 10.01	-0.3006 to 0.2236	2.123 to 17.89	44.24 to +infinity	0.004001	13.65	0.0924	1, 23	0.7639	Not Significant
